# Prescription denied: an audit of functional access barriers in California's Medi-Cal Rx system

**DOI:** 10.1093/haschl/qxag031

**Published:** 2026-02-05

**Authors:** Samah Khan, Joyce Moon Howard

**Affiliations:** Department of Social and Behavioral Sciences, New York University School of Global Public Health, New York, NY 10003, United States; Department of Social and Behavioral Sciences, New York University School of Global Public Health, New York, NY 10003, United States

## Introduction

California's 2022 consolidation of Medi-Cal pharmacy benefits into the Medi-Cal Rx program promised “simplified, standardized, and expanded” access for 14 million low-income beneficiaries via a unified state directory.^1^ This study conducted an independent audit of this directory in Fresno, California, a major urban center in a high-disparity region with high Medi-Cal concentration.

## Methods

In September 2025, we performed a cross-sectional audit of all 95 unique pharmacy listings within a 10-mile radius of Fresno (93 701) from the publicly available Medi-Cal Rx directory. A single researcher contacted each pharmacy via telephone using a standardized patient script: “Hello, I’m calling from a public health research study. Can you confirm if you are currently accepting new patients with Medi-Cal for prescription medications?” For CVS pharmacies (*n* = 22), which were universally inaccessible via the patient pathway, a secondary audit used the dedicated provider telephone line. Listings were categorically coded as: Accurate (confirmed acceptance), Limited Access (in-network but imposing restrictions or functionally unreachable), or Phantom (closed, disconnected, or not a retail pharmacy).

Following the audit, we performed data triangulation, cross-referencing all 95 listings with Google Maps (accessed January 2026) to assess the accuracy of publicly available business information. We recorded operational status, phone number congruence, and noted discrepancies to determine the locus of information failure.

## Results

### Audit of Medi-Cal Rx directory listings

Of 95 listings, only 29 (30.5%) were Accurate via the patient pathway. The majority, 66 (69.5%), presented barriers: 55 (57.9%) were Limited Access and 11 (11.6%) were Phantom (Table 1). The primary barrier was explicit refusal to dispense controlled substances to new Medi-Cal patients (eg, “not for pain medication”; “can’t take them”).

**Table 1.** Results of Medi-Cal Rx pharmacy directory audit, Fresno, CA (*N* = 95 listings).

Category	Number	Percent	Notes
Total listings	95	100%	
Accurate (initial pathway)	29	30.5%	Confirmed acceptance
Limited Access	55	57.9%	In-network but with restrictions or barriers
*Controlled Substance Refusal*	*46*	*48.4%*	“*We do accept Medi-Cal just not for pain medication” (Grizzly Pharmacy)*
*Functional Barriers*	*9*	*9.5%*	*>30 in hold, restricted call hours (M-F 9-5), automated blocks*
Phantom listings	11	11.6%	Not operational public retail pharmacies
*Permanently closed*	*5*	*5.3%*	*Calls revealed closure; prescriptions transferred*
*Institutional/nonretail*	*4*	*4.2%*	*“Only service[s] long-term care” (PharMerica)*
*Nonfunctional contact*	*2*	*2.1%*	*Immediate hang-up; number disconnected*
Accurate (CVS provider pathway)	+19	N/A	19 of 22 CVS locations confirmed acceptance via provider line
Final accessible (total pathways)	48	50.5%	

Italicized terms are sub-categories of the main audit classifications.

### Triangulation reveals systemic information chaos

Cross-referencing the directory with Google Maps revealed a deeper crisis: a systemic breakdown in public information infrastructure that extends beyond state data. The Medi-Cal directory contained unique, critical failures not present on Google. For instance, the listing for Brooks Health Care directed callers to a screeching, disconnected tone, which our investigation revealed was a fax number. Herndon Pharmacy's listed number resulted in an immediate hang-up, while Google provided a different, functional contact. However, Google Maps was also profoundly unreliable. It listed PharMerica, a pharmacy servicing only long-term care facilities, as “Open 24 hours,” actively misleading the public. For the 11 phantom listings, Google was inaccurate for 5 (45.5%). Most critically, neither platform captured the real-world rationing documented in our audit. A patient consulting Google for Pill Box Pharmacy would see an active business with no indication of its stated policy: “we can’t take [controlled substances]… we don’t even have enough for OUR patients.” This triangulation demonstrates that beneficiaries face a dual burden: a state-sanctioned directory rife with fundamental errors, and a complete absence of any reliable public resource to report real-time pharmacy capacity or restrictions.

## Discussion

Our audit reveals California's Medi-Cal Rx directory creates a “phantom network” (listings that are inaccurate or inaccessible) with widespread informal rationing of controlled substances and functional barriers like complex phone systems. The triangulation analysis shows this problem is rooted in a systemic failure of public health information infrastructure.

The directory is not uniquely flawed; it exemplifies a broader ecosystem where no reliable, real-time source of pharmacy data exists. When state directories list fax numbers and Google mislabels closed-door facilities, the navigation burden falls entirely on patients, making access a game of chance.

Policy solutions must address both curation and creation: mandating rigorous, audited state directories and requiring pharmacies to report real-time capacity and restrictions to a centralized, public-facing platform. The state must investigate pharmacy restrictions on controlled substances and consider equitable reimbursement models to disincentivize ad-hoc rationing. Regular random audits, like the one presented here, should be institutionalized as a component of regulatory enforcement.

## Conclusion

The Medi-Cal Rx directory in its current state creates an illusion of access masking reality. The phantom pharmacies and forbidden prescriptions documented in Fresno represent real-world barriers for millions of Californians. For the program's promise to be realized, the state must transform its directory from a passive, inaccurate list into an active, reliable, and equitable resource.

## Supplementary Material

qxag031_Supplementary_Data

## References

[1] Medi-Cal Rx . California Department of Health Care Services. Accessed November 2025. https://www.dhcs.ca.gov/provgovpart/pharmacy/Pages/Medi-Cal RX.aspx

